# Optimizing critical parameters for the directly measurement of particle flow with PF-SIBS

**DOI:** 10.1038/s41598-018-20073-1

**Published:** 2018-01-30

**Authors:** Shunchun Yao, Jialong Xu, Lifeng Zhang, Jingbo Zhao, Zhimin Lu

**Affiliations:** 1 School of Electric Power, South China University of Technology, Guangzhou, Guangdong, 510640 China; 2 Guangdong Province Key Laboratory of Efficient and Clean Energy Utilization, Guangzhou, Guangdong, 510640 China; 3 Guangdong Province Engineering Research Center of High Efficiency and Low Pollution Energy Conversion, Guangzhou, 501640 China

## Abstract

A novel measurement technology named as particle flow-spark induced breakdown spectroscopy (PF-SIBS) was reported for real-time measurement of solid materials. Critical measurement parameters of PF-SIBS were optimized and a set of fly ashes with different carbon content were measured for evaluation of measurement performance. Four electrode materials, tungsten, copper, molybdenum and platinum, were compared in the aspects of signal stability, line interference and electrode durability. Less line interference and better signal stability were obtained with W and Cu electrode, while W electrode has better durability. Quartz sand with diameters from 48 μm to 180 μm were tested to investigate the influence of particle size. As the particle diameter increased, the intensity of Si 288.16 nm line decreased while that of ambient air constituents increased. To reduce the particle effect, the sum intensity from sample and ambient air were introduced to correct. The RSD of line intensity between the five diameters were reduced from 67.30% to 16.59% with Cu electrodes and from 63.21% to 13.64% with W electrodes. With the optimal measurement parameters and correction, fly ash samples with different carbon content were tested and the correlation coefficients R^2^ of multivariate calibration achieved 0.987.

## Introduction

The detection of particle materials represents a major aspect of analytical needs for they are widely present in the industrial production and emission process. Thus, the real-time measurement of constituents in particle materials would be favor for achieving optimal operation and controlling pollution emission. Composition measurement can be performed via standard analysis techniques with good sensitivity and low detection limit, such as inductively coupled plasma-atomic emission spectroscopy (ICP-AES)^1^, inductively coupled plasma-mass spectrometry (ICP-MS)^2^ and X-ray fluorescence (XRF)^3^, while the preparation process is time-consuming and not practical for field application. Laser induced breakdown spectroscopy (LIBS) is an emerging on-line measurement technique for its simple or no sample treatment, rapid response and multi-element analysis. Several researches about real-time measurement of particle materials has been conducted^4–6^. However, high investment and poor durability of laser instrument limit LIBS to widely apply in some industrial field. To produce plasma for analysis with cost-effective and robust equipment, spark induced breakdown spectroscopy (SIBS) has been proposed. The plasma is produced by an electric discharge spark between two electrodes to excite samples, thus the instrument can be more compact and field-portable without the need of laser beam alignment. The potential of applying SIBS for real time measurement has been discussed in much research. The study of Kawahara *et al*.^7^ illustrated that SIBS can be a diagnostic tool for spark-ignition engines and determined the local equivalence ratio of a CH_4_/air mixture in a laminar premixed flame. Taefi *et al*.^8^ used SIBS for qualitative analysis of cement powder and both major and minor elements can be detected. The calibration results show the possibility of SIBS applied in industry for on line analysis of powder samples. Schmidt *et al*.^9^ demonstrated the good linear dependence between the temporally integrated feature strength in SIBS and elemental compositions of three bioaerosol samples. And the phenomenon of varying decay kinetics from sample to sample has been observed. The authors also applied SIBS for monitoring changes in the concentration of carbon in soil^10^. Good correlation between the predicted and measured carbon concentrations were obtained by using PLS regression analysis. Khalaji *et al*.^11^ studied the potential of SIBS for continuous dust monitoring and found that it has enough fastness and sensitivity for detect variation in dust level due to normal foot traffic inside a room. Diwakar *et al*.^12^ developed an aerosol preconcentration system coupled with SIBS. The charged aerosol particles were collected onto the flat tip of an electrode by electrostatic deposition and then analyzed by SIBS, and excellent repeatability and detection limits of spectral measurements were obtained in the system. Their group also applied the system for near real-time measurement of carbonaceous aerosol^13^. They established a single calibration curve by pooling together the data for all the organic and inorganic carbonaceous materials, and the measurement results agreed well with the thermal optical method.

The above study has demonstrated that SIBS is applicable to the rapid measurement of constituents in solid materials, and the common measuring states of particle samples for analysis are pelleting and packing. However, ablation and breakdown of pelleted materials with high mechanical strength need higher energy and thus result in higher background emission from electrodes. To reduce the spark energy needed for ablation, laser beam was introduced to ablate sample and produce original plasma in previous studies^14,15^. However, it is contrary to our pursuit of compact and low-cost instrument. Another treatment method is putting the electrodes into or above the surface of particle samples in packed state, while the ejection of packed samples due to the shock wave would influence the optical measurement. By combining SIBS and particle flow, we proposed the route for direct analysis of particle sample and named it as PF-SIBS. Without the sample pretreatment process for powder samples, the method is of benefit to continuous measurement in industrial field. To further develop the application of PF-SIBS for real-time measurement, the fundamental parameters of PF-SIBS is significant and it is studied specifically in this work for optimal measurement results. These fundamental parameters can be broadly divided into two parts: the emission characteristic of spark plasma and the influence of particle size. The emission of spark plasma is closely related to the electrodes as the plasma generates from the electrode discharge. In order to produce stable plasma, electrode should have the characteristics of excellent erosion-resistance, high electrical conductivity and high thermal conductivity. Furthermore, as the erosion of electrodes is inevitable due to the hot plasma between them, the spectral line of electrode materials might influence the detection of analyte and should be taken into consideration. The selection of electrode materials is of significance to the accurate measurement and there are no specific research about it so far. Molybdenum^16^, platinum^17^, copper^18,19^ and tungsten^10–13^ were chosen as the electrode materials for experiment as they are the common electrode materials, and the spectral interference, plasma characteristic, delay time were studied to obtain optimal plasma observation state. Different size of quartz sands were also tested to investigate the size effect. Finally, after optimizing the measurement parameters, eleven fly ash samples with different carbon content were tested for preliminary evaluation of the feasibility of PF-SIBS.

## Results and Discussion

### Emission characteristics of different electrode materials

**Figure 1:**
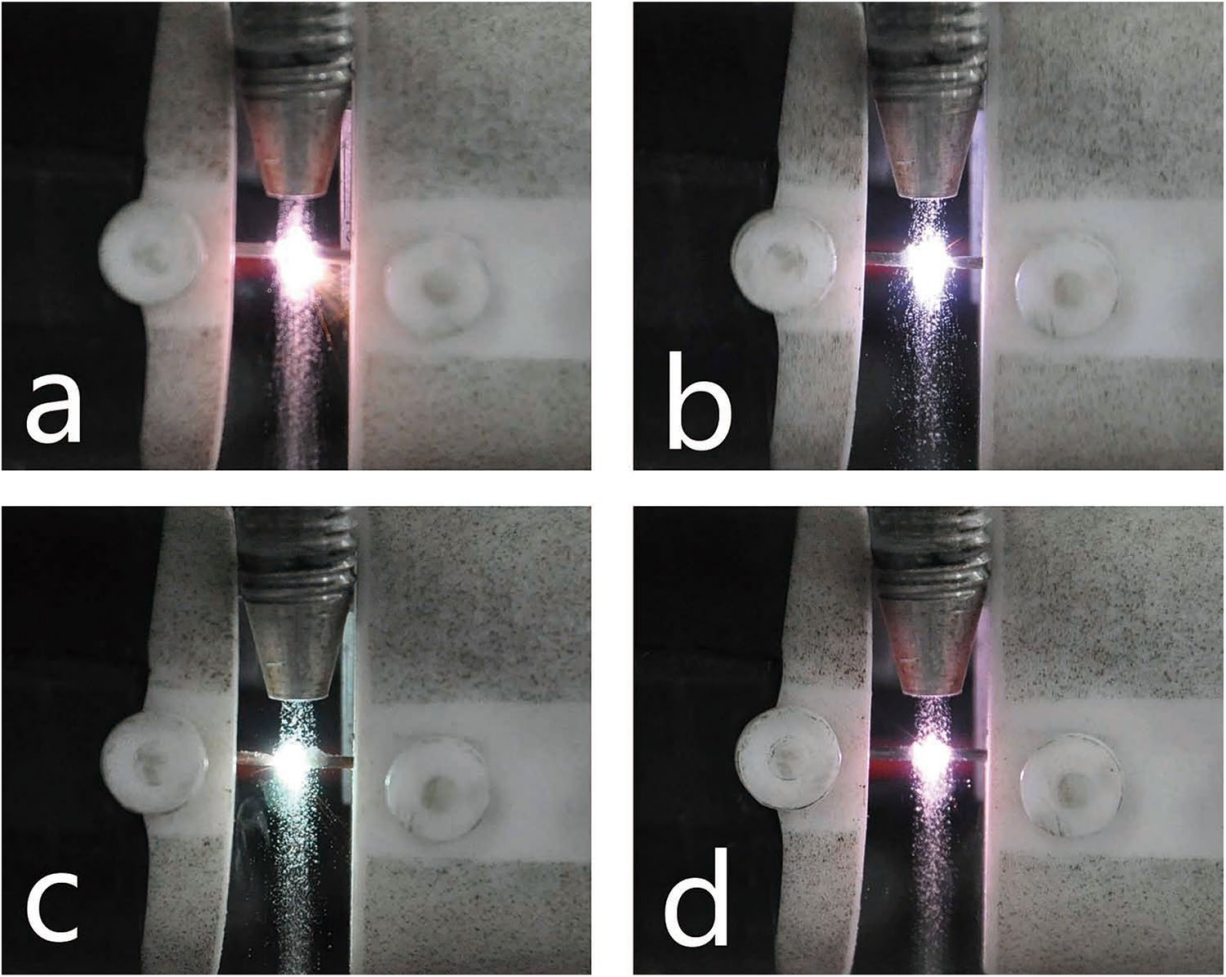
Optical breakdown induced in particle flow with different electrode materials: (**a**) Mo; (**b**) Pt; (**c**) Cu; (**d**) W.

**Figure 2:**
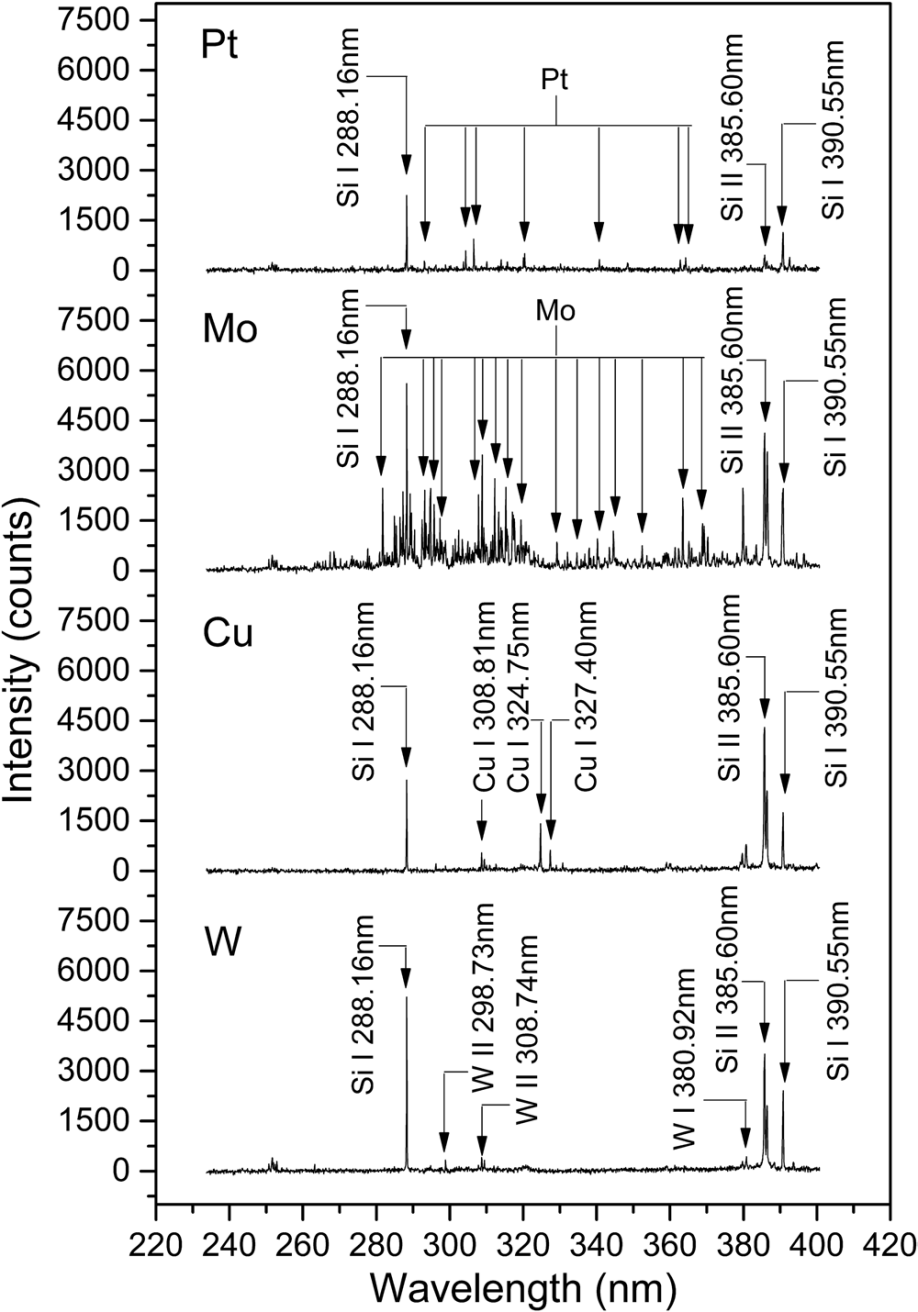
Spectra of quartz sands with different electrode materials.

To investigate the emission characteristics of spark plasma with different electrode materials, four selected electrode materials were applied to detect quartz sands in the same experiment condition. The optical breakdown induced in particle flow of quartz sands was recorded by a camera (Nikon, D90, Japan) and illustrated in Fig. 1. The plasma size were similar with the same spark gap and voltage, while the plasma color are different because of the different emission from electrode materials, which can demonstrate the influences of electrode materials to spectroscopy analysis. The spectra of quartz sands with different electrode materials are shown in Fig. 2. The spectrum of Pt electrode were lower than others and the plasma breakdown were found to be instable during the experiment. The instable breakdown phenomenon is related to the physical characteristics of Pt (shown in Table 1), its highest ionization energy, electrical resistivity and lowest thermal conductivity in four selected materials have adverse effects to the ionization process before breakdown and thus affect the following plasma formation. Therefore, Pt electrodes are inappropriate for the PF-SIBS to obtain continuous stable measurement. When particle flow were detected by Mo electrodes, stable plasma could be generated while much electrode line interferences were also observed from the spectrum (shown in Fig. 2). It is resulted from the characteristics of low ionization energy and high transition probability of Mo. Although Mo electrode can create stable air plasma, it is still not a suitable electrode material for PF-SIBS because these serious line interferences will affect the accurate detection of complex samples and it is hard to be corrected. The results showed that Cu and W are two electrode materials which can produce stable plasma with fewer line interferences. Considering the relatively low melting point of Cu, the erosion of electrodes would be more than W. Therefore, W electrode is more applicable for long-term operation to reduce the need for electrode replacement. In short-term measurement, both the two electrodes (W and Cu) could be used and the performance of this two electrodes would be investigated in the flowing study.

**Table 1:** Physical characteristics of electrode material.

	Mo	Pt	Cu	W
Ionization Energy (ev)	7.09	8.96	7.73	7.86
Electrical Resistivity-27 °C(Ω·cm)	5.70E-06	1.06E-05	1.70E-06	5.65E-06
Melting Point (°C)	2617	1769	1083	3370
Thermal Conductivity-27 °C (W·m^−1^·K^−1^)	138	69	398	163

**Figure 3:**
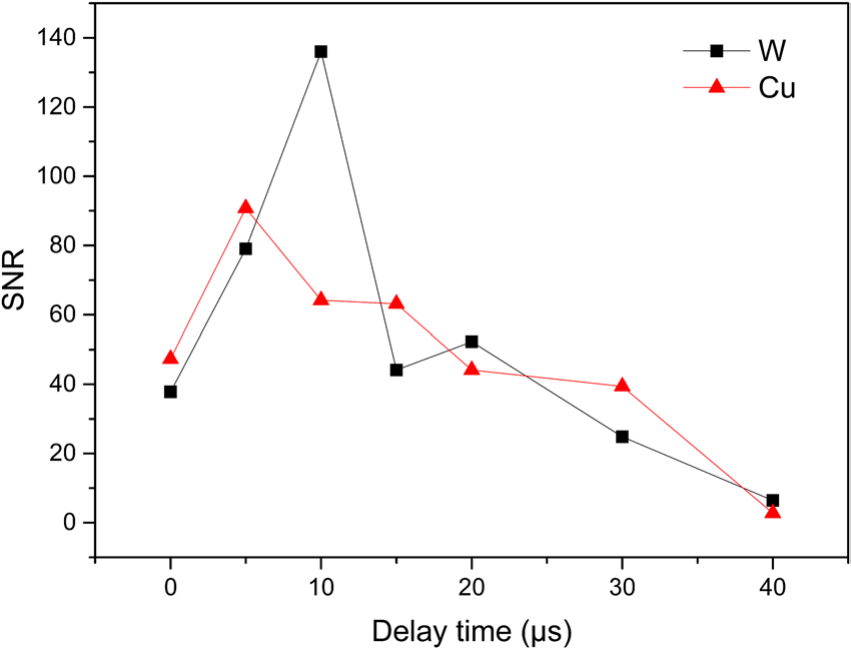
Variation of SNR of Si 288.16 nm with different delay time.

To avoid the influence of background continuum on spectral analysis at early times, delay time is a key measurement parameters to be optimized. As the background continuum decays with time more quickly than characteristics spectral line, there would be an optimal time for spectral line detection and signal to noise ratio (SNR) was used to determine it. In this study, Si 288.16 nm line was selected as feature line for calculating SNR. As shown in Fig. 3, the evolution time of Si 288.16 nm line can reach 40 μs, they are much longer than the plasma evolution time in LIBS. The optimal delay time was determined to be 5 μs with Cu electrode and 10 μs with W electrode, which would be used in the following experiment and better SNR was observed with W electrode.

### Size effect of particle flow in PF-SIBS

Particle size inhomogeneity is one of the major matrix effects in the measurement of particle flow. The influence can be broadly classified into two types: (1) the uneven distribution of element depended on the particle diameter;^20,21^ (2) the size effect to the plasma–particle interaction^22,23^. In this paper, to evaluate the size effect in spark plasma, quartz sands with different diameters were tested to exclude influence of compositional dependence on the particle diameter. Similar to the analysis of particle flow by LIBS, partial breakdown spectra might be observed in the case of PF-SIBS because the number density and distribution of particles in the plasma volume are varied with the fluctuation of particle flow. Conditional data analysis has been proposed in previous publication for processing the spectra and signal-to-noise ratio (SNR) was set as index to identify the partial breakdown spectra^24^. With the threshold value of the SNR (SNR = 4) of Si 288.16 nm, all the spectra from PF-SIBS with different diameter were identified as true spectra in the experiment, while around 10% spectra from LIBS were identified as partial breakdown spectra in our previous study^25^. The effect of particle diameter on partial breakdown was lower in PF-SIBS for the reason that the plasma volume of SIBS is larger than LIBS. The diameter of plasma volume of SIBS reaches several millimeters while that of LIBS is on the order of 0.1 mm^26^. Therefore, PF-SIBS can process larger number and size particles in particle flow, resulting in a more representative sampling in different diameter.

**Figure 4:**
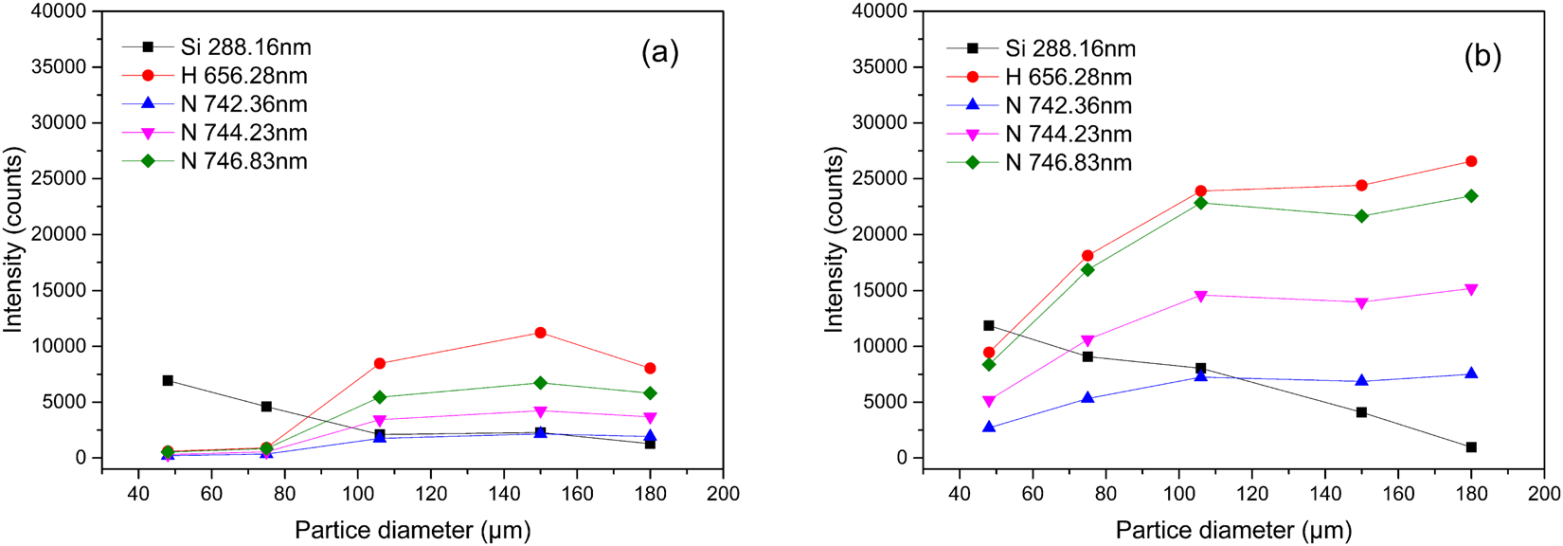
Variation of line intensity with particle diameter: (**a**) Cu electrode; (**b**) W electrode.

Another influences of size effect is the variation of emission intensity with different particle diameter. As illustrated in Fig. 4, the line intensity of Si 288.16 nm decreased with the particle diameter increased, while the intensity of spectral line emitted from ambient air also went up. It is because the increase of particle diameter with same mass flow made the specific surface area of particles become smaller, which would lower the vaporization and dissociation efficiencies in plasma–particle interaction and eventually led to the decrease of spectral line intensity from particle samples. In the meantime, as the energy of spark plasma were relatively steady after breakdown, more plasma energy would be used to excite ambient air and caused the increase of line intensity of them.

**Figure 5:**
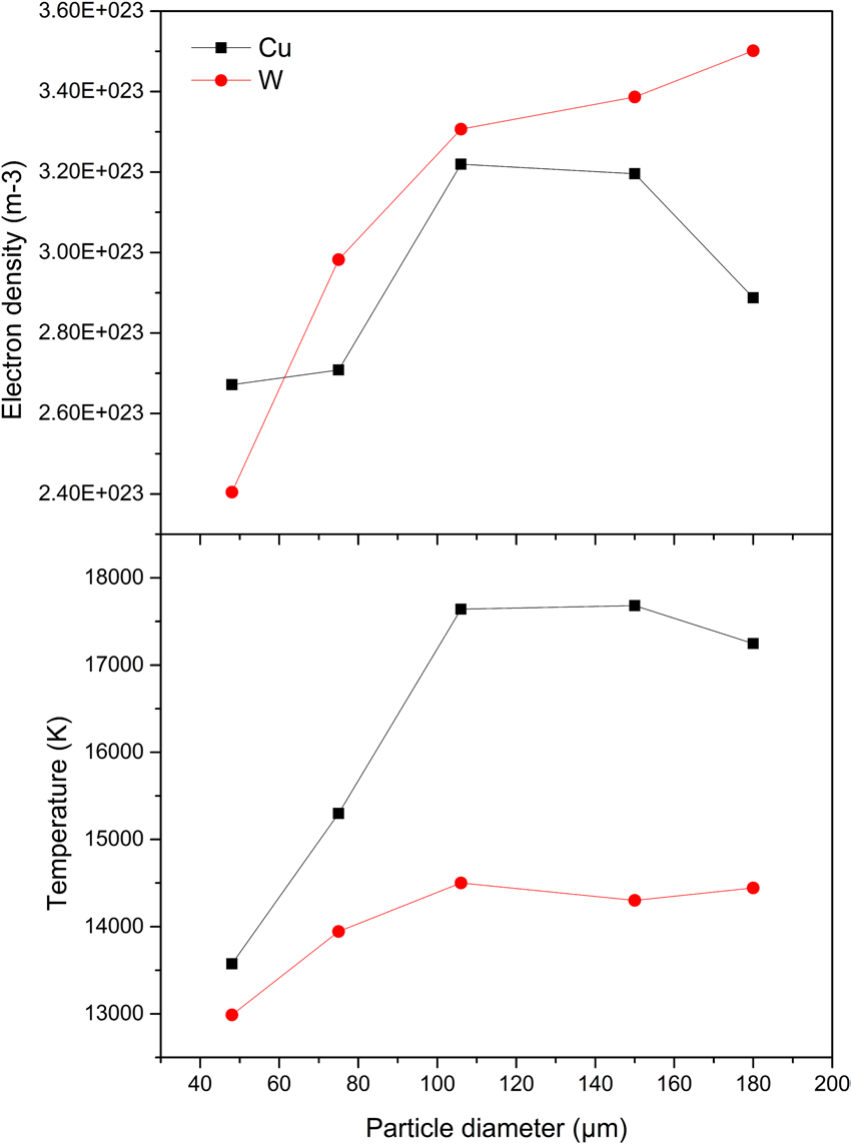
Variation of plasma parameters with particle diameter: (**a**) electron density; (**b**) temperature.

To further investigate the cause of the emission intensity variation, the electron density and plasma temperature were calculated. Electron density was obtained using Hα line at 656 nm and then used for plasma temperature calculation by Saha–Boltzmann plot^27^. Saha-Boltzmann plot method can provide an accurate estimation of plasma temperature by a wider range of the upper level energies from the neutral and ion species of the same element. As the unintensified CCD were used in this experiment in order to to reduce costs of the measure system, the acquisition time is long. Therefore, the plasma temperature and electron density were calculated as average index in the whole plasma life rather than accurate value to evaluate the size effect in spark plasma^28^. The list of spectral lines parameters used for calculation is summarized in Table 2. Using these lines, the electron density and temperature of plasma were obtained (shown in Fig. 5) and they were increased with the rise of particle diameter.

**Table 2:** List of spectral lines used for temperature calculation.

	λ_ki_(nm)	E_k_(eV)	g_k_A_ki_(10^8^s^−1^)
Si I	288.16	5.08	6.51
Si I	390.55	5.08	0.40
Si II	634.71	10.07	2.34
Si II	637.14	10.06	1.36

These above changes of intensity, electron density and temperature are attributed to the plasma–particle interaction with different particle size. As discussed in earlier studies^13^, the spark formation in air take places from the electrode discharge. A high voltage pulse is first applied between electrodes and a conducting ion channel is formed to induce dielectric breakdown of air. Then a finite amount of energy is deposited into the spark gap and the plasma evolved. The energy of plasma is finite and its distribution is of significance to be studied, which is related to the plasma–particle interaction. When particles pass through the plasma, the physical processes in the interaction contains vaporization, dissociation, heat and mass transfer, ionization, electronic excitation and radiative transfer^29^. In an ideal scenario, the time scales of these physical processes are instantaneous, thus the analyte species can be dissociated completely and the signal would be largely independent of particle size at the same mass flow. However, in practice, the mass dissociation rate are finite relative to the detection time and thus will vary with the surface-to-volume ratio. The introduction of mass for detection will be different for the incomplete vaporization and dissociation at the same mass flow. With the different mass dissociation, the mass transferred from the particles to the plasma and the heat transferred from the plasma to the particle are also different. In this study, with the particle diameter increased at the same mass flow, the ablation efficiency decreased and less heat was consumed by evaporation and dissociation of particles, leading to the rise of temperature. Moreover, as less plasma energy was used to dissociate the analyte, more energy would be used for electronic excitation and improved the excitation degree, manifesting in the increase of electron density, which is also related to the increase of temperature.

With the above study of the plasma characterization, the correction method of the spectra line intensity variation from different diameter samples was proposed. To evaluate the spectra line intensity variation, the relative standard deviation (RSD) of Si 288.16 nm line intensity between the five diameters was introduced. It was 67.30% with Cu electrode and 63.21% with W electrode. As the variation of the line intensity from particle samples and that from ambient air showed opposite trend, the sum of line intensity form particles and ambient air were used to compensate for variation because of the plasma energy conservation^30,31^. Nitrogen is the main constituent of ambient air and thus it was chosen for the compensation. By combining the line intensity of Si 288.16 nm and N 744.23 nm, the RSD of the sum intensity can reduced to 16.59% with Cu electrode and 13.64% with W electrode, which is relatively stable under the influence of size effect.

**Figure 6:**
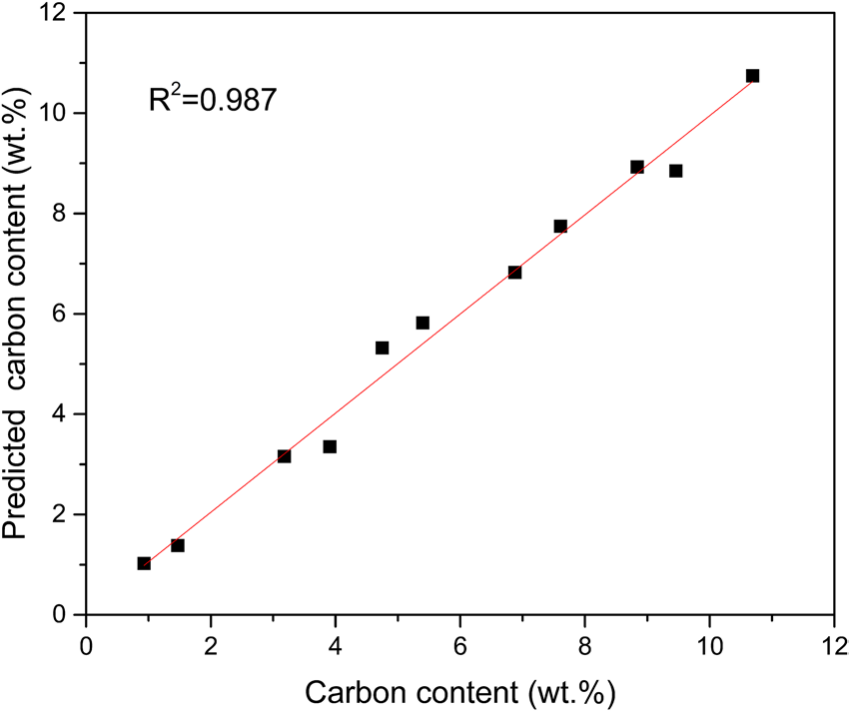
Calibration curves for carbon content of fly ash in PF-SIBS.

To preliminary investigate the measurement effect of PF-SIBS after optimizing the measurement parameters, seven fly ash samples taken from coal fired power plant with different carbon content were tested with tungsten electrode. The carbon content in the fly ashes was determined by the loss-on-ignition (LOI) method^32^ and listed in Table 3. The intensity ratios of C 247.86 nm, Al 256.8 nm and Ca 393.37 nm to Si 288.26 nm combined with the above particle size effect correction were selected as independent variables in the linear multivariate calibration models, and the correlation coefficient R^2^ of the calibration model achieve 0.987 (shown in Fig. 6), which is acceptable in the preliminary investigation. The results indicated the feasibility of PF-SIBS for quantitative analysis and it would be discussed in our further study.

**Table 3:** Carbon content of fly ashes.

	1#	2#	3#	4#	5#	6#	7#	8#	9#	10#	11#
Carbon content(%)	0.93	1.47	3.18	3.91	4.75	5.40	6.88	7.61	8.84	9.46	10.69

## Conclusions

Several measurement parameters of PF-SIBS were optimized. Firstly, the emission characteristics with four electrode materials in PF-SIBS were compared, the electrode of Cu and W performed better than Mo and Pt for less line interference and better plasma intensity. Optimal delay time of Si 288.16 nm line in Cu and W electrode were observed as 5 μs and 10 μs. Size effect of particle samples were then investigated and five diameter quartz sands were tested. The influence of size effect to partial breakdown were not observed in PF-SIBS for its bigger plasma volume, while the influence to the line intensity of sample constituents was obvious. The line intensity of Si 288.16 nm increased with the particle diameters increased and the RSD of intensity between the five diameters was 67.30% with Cu electrode and 63.21% with W electrode. After studying the characteristics of plasma-particle interaction in PF-SIBS, N 744.23 nm line intensity was applied to compensate the variation of Si line as their trend of the change were opposite, and the RSD was reduced to 16.59% with Cu electrode and 13.64% with W electrode. Fly ash with different carbon content were tested to investigate the measurement effect and acceptable correlation coefficient (R ^2^ = 0.987) was obtained. These results suggest that PF-SIBS is a feasible method for real-time measurement of particle samples with suitable electrode and the influence of size effect on line intensity can be compensated. The Cu and W are proved to be suitable electrode materials while W is more in favor of long-term operation for its high melting point. Larger size and more stable position of plasma between two electrodes in PF-SIBS can reduce the influence of size effect and particle flow fluctuation to partial breakdown. However, the size effect to emission of particle constituents still exists, and it can be reduced by combining the emission of ambient air or further controlling the size distribution of particle flow to obtain accurate analysis results.

## Methods

**Figure 7:**
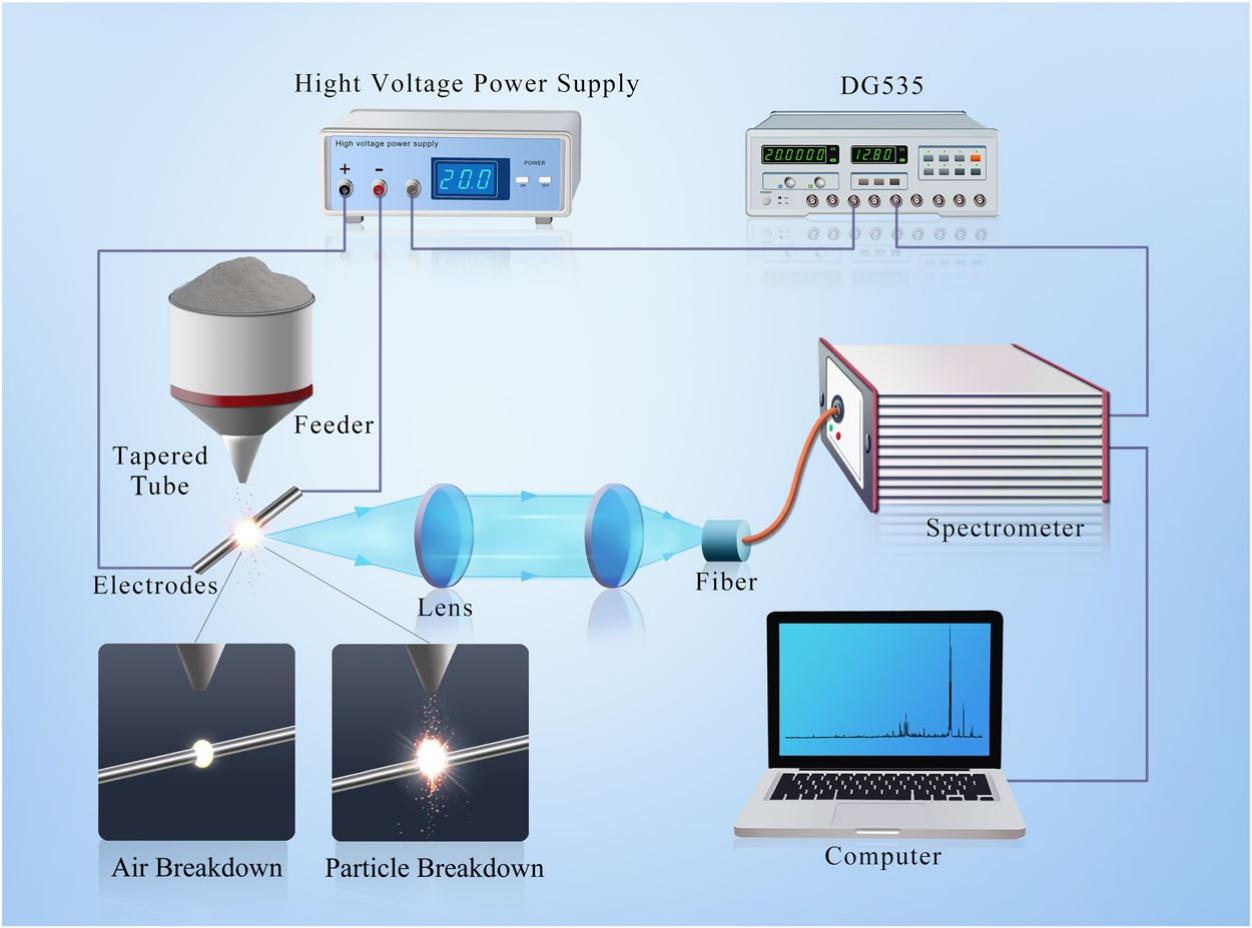
Schematic diagram of the PF-SIBS system.

As schematically illustrated in Fig. 7, the PF-SIBS experimental setup used to measure the particle flow consists of three units: a particle flow generation system, a spark generator system and a spectroscopy detection system. Particle flow was generated using a piezoelectric type vibrational feeder (PEF-90A, Sanki, Japan) with a tapered tube, which is used to enrich the particles and reduce the flow fluctuation. Then the particles passed through the spark gap with a mass flow maintained at 1.2 g/min throughout the experiment. In the spark gap, air plasma was generated between two 1 mm coaxial electrodes with conic shaped tip by a high voltage pulse from the voltage power supply (4.5 kV, DC), the distance between electrodes was 1 mm for stable discharges. As the particles were ablated and vaporized by the air plasma, characteristic spectra from the samples were emitted and then collected and transmitted to a spectrometer (AvaSpec-2048, Avantes, Holland) by an optical fiber for analysis. The dual channel spectrometer covers a wavelength range from 235 to 400 nm and 575 to 790 nm, with a spectral resolution of 0.1 nm. Moreover, the spectrometer was synchronized with the high voltage power supply by a delay generator (DG535, Stanford Research Systems, America) to adjust the pulse frequency and delay time.

Mo, Pt, Cu and W of 99.9% purity were selected as the electrode materials in the experiment. To avoid the interference of matrix effect from complex samples composition, quartz sands were selected as experiment samples. Five diameters samples were prepared: 180, 150, 106, 75, 45 μm, which are among the approximately distribution of particle sizes in practice.
